# Thermal Degradation of Carboxymethyl Cellulose (CMC) in Saline Solution for Applications in Petroleum Industry Fluids

**DOI:** 10.3390/polym17152085

**Published:** 2025-07-30

**Authors:** Mirele Costa da Silva Farias, Waleska Rodrigues Pontes da Costa, Karine Castro Nóbrega, Victória Bezerra Romualdo, Anna Carolina Amorim Costa, Renalle Cristina Alves de Medeiros Nascimento, Luciana Viana Amorim

**Affiliations:** 1 Laboratório de Pesquisa em Fluidos de Perfuração (PEFLAB), Universidade Federal de Campina Grand (UFCG), Rua Aprigio Veloso, 882, Bairro Universitário, Campina Grande 58429-900, PB, Brazil; mirelecsilva@hotmail.com (M.C.d.S.F.); karine.nobrega@hotmail.com (K.C.N.); vivi.victoria07@gmail.com (V.B.R.); anna.amorimc@gmail.com (A.C.A.C.); luciana@uaepetro.ufcg.edu.br (L.V.A.); 2 Unidade Acadêmica de Santo Agostinho, Universidade Federal Rural de Pernambuco (UFRPE), Rua Cento e Sessenta e Três, 300, Garapu, Cabo de Santo Agostinho 54518-430, PE, Brazil; renalle.nascimento@ufrpe.br

**Keywords:** carboxymethyl cellulose, rheological behavior, thermal analysis, brine, thermal aging, polymer degradation

## Abstract

This work aims to evaluate the thermal degradation of carboxymethyl cellulose (CMC) in solution in the presence of salt, as well as to study the correlation of the rheological behavior of these solutions with exposure to temperature. Step 1 involved characterizing powdered low- and high-viscosity CMC using SEM, FTIR, TGA/DrTGA, and DSC. In step 2, CMC solutions in fresh and saline water were characterized by TGA/DrTGA and viscosity tests. Step 3 exposed saline solutions to 70–150 °C for varying times, followed by TGA/DrTGA and viscosity analyses. There were no significant differences in the thermal degradation of LV and HV CMC, nor in terms of the physical state of the polymer. The results demonstrate that the use of CMC necessitates a combined analysis of thermal degradation and rheological behavior.

## 1. Introduction

By 2050, there will be an estimated 9.8 billion people on Earth, a massive population increase that will require an ever-increasing supply of energy. Global oil and gas production can help meet this demand for the foreseeable future [1]. This issue brings the need for drilling more complex wells, thus increasing the demand for more efficient exploration of productive formations [2].

On the one hand, population growth drives increased fossil fuel demand, resulting in a surge in oil exploration and production. However, the ongoing collaboration between science and industry to adopt cleaner energy sources may lead to the decommissioning of oil facilities and the abandonment of oil wells.

In the context of the oil industry, fluids are present in drilling, completion, production, and well abandonment operations [3,4,5,6]. The effective operational performance of these activities is closely linked to the careful selection of the fluid [7]. This selection depends on the use of optimized formulations, which take into account the choice of components and their proportions, as well as the specific operational conditions of each well, such as temperature.

In fluid formulation, filtration reducers and rheological modifiers are essential additives that enhance fluid characteristics and performance [8]. Examples of these additives include xanthan gum, agar-agar, hydroxyethyl cellulose, gums, carboxymethyl cellulose (CMC), and other biopolymers [7,9,10,11,12,13,14]. Among these additives, CMC clearly outperforms the others mentioned [11]. It has established itself as the leading choice in drilling fluids [15].

Carboxymethyl cellulose is an anionic hydrophilic colloid [16], soluble in water, and with a linear anhydroglucose structure [17]. It is synthesized by chemical modification through carboxymethylation of cellulose from its heterogeneous reaction with monochloroacetic acid (etherifying agent) under excess sodium hydroxide (swelling agent), thus resulting in the partial replacement of -OH groups of glucose in positions O-2, O-3, or O-6 by the carboxymethyl radical [18,19].

Characteristics such as non-toxicity, biodegradability, and biocompatibility make carboxymethyl cellulose one of the most important cellulose derivatives [20]. In addition to properties such as thickening, binding, emulsifying, stabilizing [21], and good mechanical strength [22], its high surface hydration capacity, strong interaction with bentonite, and the presence of a complex structural network allow CMC to act as an efficient rheological modifier. Additionally, its nanometric dimensions and easily adjustable surface chemistry make it suitable as an effective reducer of filtrate loss [11]. These characteristics make carboxymethyl cellulose an essential additive in fluid formulations.

Although CMC offers several advantages, it is susceptible, for example, to thermal variations and critical pH changes, which implies limitations related to its synthesis and application [23]. Regarding the use of CMC in fluids, CMC undergoes hydrolysis of the polymer chain when subjected to high-temperature conditions in an aqueous medium, resulting in the rupture of the chain and compromising the properties of the fluids, since the molar mass of polymers is directly related to their properties [24].

Considering the wide range of thermal stresses that fluids in the oil industry must withstand, it is essential to emphasize the important role of carboxymethyl cellulose (CMC) as a rheological agent in the formulation of aqueous fluids. However, the thermal degradation of CMC has been extensively studied mainly in its powder form, or in solutions prepared only with fresh water. As a result, most published studies do not accurately replicate the conditions to which CMC is subjected in these industrial fluids, which commonly involve saline media.

This study aims to evaluate the thermal degradation of carboxymethyl cellulose in solution in the presence of salt, as well as to investigate the correlation of the rheological behavior of these solutions with exposure to temperature.

## 2. Materials and Methods

### 2.1. Materials

In this work, two samples of carboxymethyl cellulose of purified grade, (Denver Especialidades Químicas LTDA, Cotia, Brazil) were used. The first sample presented low viscosity (95 cP) and a degree of substitution (DS) of 0.75. The other sample presented high viscosity (507 cP), with a degree of substitution (DS) of 0.82. These samples were named CMC LV and CMC HV, respectively. In the first stage of the work, the CMC samples were used separately, in the physical state of powder.

For step 2, two solutions were prepared using the polymers CMC LV (4.17 g/100 mL) and CMC HV (0.26 g/100 mL). For one of the solutions, named SFW, the dispersing phase used was composed only of fresh water. For the second type of solution (SSW), saturated NaCl brine (36 g/100 mL) was added, in addition to fresh water. In step 3, only the SSW solution was used for the study. The concentration of the solutions was based on formulations of fluids used in the petroleum industry.

Combining LV and HV CMCs in solution aims to meet the needs of the oil industry, since the fluids, in most cases, need to simultaneously present low filtrate volume (a property provided by CMC LV) and adequate viscosity to avoid sedimentation (provided by CMC HV). These properties are closely interconnected, since the increase in fluid viscosity contributes to the reduction in filtrate loss. Thus, the rheological behavior of CMC solutions was monitored in this study, using the procedures described in the following topic, in order to investigate the efficiency of the polymers in controlling viscosity and, indirectly, in reducing filtrate, under the conditions analyzed.

### 2.2. Methods

Table 1 shows the identification of the samples, as well as their physical state and tests performed at each stage of this study.

#### 2.2.1. Characterization of CMC Powders

The CMC powders were morphologically characterized by SEM, using a S5123 scanning electron microscope (Tescan, Brno, Czech Republic) operating at 10 kV. For chemical characterization, the samples were analyzed by FTIR, using a Spectrum 400 spectrometer (Perkin Elmer, Waltham, MA, USA). The analyses were performed with 25 scans in the spectral range of 4000 to 400 cm^−1^, under a nitrogen atmosphere.

TGA/DrTGA and DSC analyses were performed with a PerkinElmer thermal analyzer.

#### 2.2.2. Characterizations of CMC Solutions at Room Temperature

SFW and SSW were prepared in a homogenizer (Hamilton Beach Brands Inc., Glen Allen, VA, USA). For SFW, initially, CMC LV (2.86 g) was added to deionized water (68.57 mL) with a stirring interval of 5 min. Then, CMC HV (0.1786 g) was incorporated, maintaining stirring for another 5 min.

The same amounts of the components of the SFW was used in the SSW. However, the polymers were initially dissolved in saturated NaCl brine (36 g/100 mL), maintaining the stirring time of 5 min after the addition of each polymer. Finally, deionized water was added to complete the volume of the solution.

For sample characterization, a PerkinElmer thermal analyzer was used, through which TGA/DrTGA and DSC analyses were performed. The samples were heated from 25 °C to 600 °C, at a heating rate of 10 °C/min, in alumina crucibles, under a dynamic nitrogen atmosphere with a flow rate of 100 mL/min.

The determination of the viscosity values at different shear rates for the solutions was performed on a Haake Mars 60 rheometer (Thermo Scientific, Karlsruhe, Germany), using a 35 mm sand-blasted parallel-plate geometry set (P35/Ti/SB). The viscosity curves were obtained by a controlled shear rate method at room temperature, using a gap value of 1 mm between the plates. The shear rate variation was performed continuously from 0.1 s^−1^ to 1000 s^−1^.

#### 2.2.3. Exposure to Temperature of Saline Solution (SSW)

The exposure conditions of the solutions were established using a factorial design, where temperature and exposure time were the variables, resulting in a total of seven experiments (2^2^ + 3 central points). The experiments were performed randomly to avoid systematic errors, and the monitored response was the loss of CMC mass in solution, obtained from the thermal analysis tests. The regression of the experimental data was performed using Statistica software, version 7.0 (Stat-Soft, Tulsa, OK, USA). Table 2 presents the factors studied and their respective levels, and Table 3 presents the planning matrix used to perform the tests.

The solutions were exposed to temperature in stainless steel cells pressurized with nitrogen gas at 100 psi, in a Fann Roller Oven.

After depressurizing the cells and cooling the solutions, samples were collected for TGA/DrTGA analysis. These analyses were performed in a DTG-60 H thermal analyzer (Shimadzu, Kyoto, Japan). The samples were heated from 25 °C to 600 °C, at a heating rate of 10 °C/min, in alumina crucibles, under a dynamic nitrogen atmosphere with a flow rate of 100 mL/min. In addition, viscosity testing was performed on a Haake Mars 60 rheometer, using the same methodology described in step 2.

## 3. Results and Discussion

### 3.1. Characterization of CMC Powders

**Figure 1:**
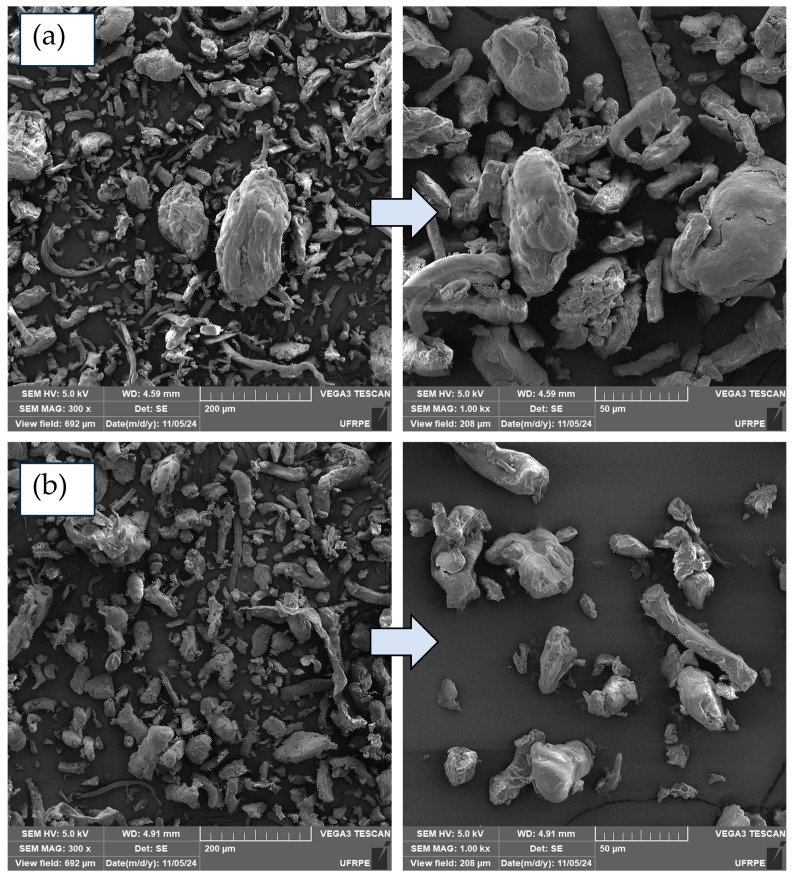
Micrographs of powder samples of CMC LV (**a**) and CMC HV (**b**), at magnifications of 300, 800, and 1000×.

Figure 1 shows the micrographs obtained by scanning electron microscopy of CMC LV and CMC HV, at different magnifications.

According to the micrographs, the morphology of CMC LV and CMC HV presents similar characteristics. The fibrous particles observed are a typical characteristic of cellulose. They are distributed heterogeneously throughout the analyzed regions, presenting variations in length and diameter, and may be isolated or form agglomerates with dimensions greater than 50 µm. These results suggest that the classification of CMC’s viscosity is more related to its chemical properties than to its physical characteristics.

**Figure 2:**
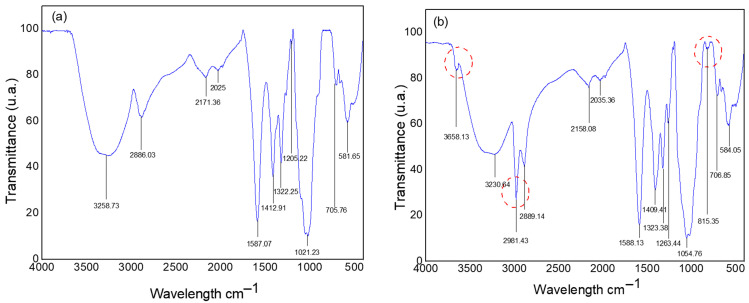
Spectra of powder samples of CMC LV (**a**) and CMC HV (**b**).

Figure 2 presents the Fourier-Transform Infrared (FTIR) spectra for the LV and HV CMC powder samples.

The spectra presented show absorption bands in different positions and intensities, varying according to the presence of functional groups associated with the CMC structure. A distinction in the chemical characteristics between CMC LV and CMC HV can be observed, as evidenced by the presence of some exclusive bands in the CMC HV spectrum. Among these, the band located at 3658 cm^−1^ refers to the presence of the alcohol functional group –OH [25], the band at 2961 cm^−1^ is attributed to the stretching vibrations of the C–H groups [26], and the band at 816 cm^−1^ corresponds to the bending of the C–H bond, resulting from a tri-substitution. The presence of variations in uniformity and crystallinity can cause different chemical interactions between the functional groups present in CMC. The crystalline arrangements of cellulose are impacted depending on the reactive functional groups. In the cellulose transformation process, four main crystalline polymorphs of cellulose are already known, and this three-dimensional arrangement affects the stability of its structure [27].

The other spectral bands are similar for both CMCs. The broad absorption band centered at approximately 3200 cm^−1^ refers to the OH stretching region of the hydrogen bond [28]. A low-intensity band appears around 2880 cm^−1^, caused by the stretching vibration of the C–H bond [29]. Another low-intensity and low-absorption band appears between 2025 and 2171 cm^−1^, characterized as weak signals corresponding to interactions between N^+^ and H^−^ [30]. At positions 1588 cm^−1^ and 1400 cm^−1^, sharp bands of high intensity are found, identifying the presence of carboxyl groups COO- [29] and typical bands for asymmetric and symmetric stretching of the carboxylate -COONa^+^ [31], respectively.

The bands at approximately 1320 cm^−1^ correspond to the bending vibration of the -OH group, while around 1200 cm^−1^ there is a band of low absorption, which characterizes the presence of the alkylaryl ether group CO or vibrational bands attributed to C–O stretching [32]. The most intense peak, representing the highest absorption for both CMCs, is located at approximately 1000 cm^−1^ and is attributed to strong C–O bonds [25]. At 705 cm^−1^, another low-absorption band appears, related to the presence of the CH_2_ group [25]. Finally, a band at 581 cm^−1^ is observed by the presence of glycosidic bonds [33]. In general, the evaluated CMCs present the same functional groups, and they may present small changes regarding the interactions between the groups mentioned in the discussion.

**Figure 3:**
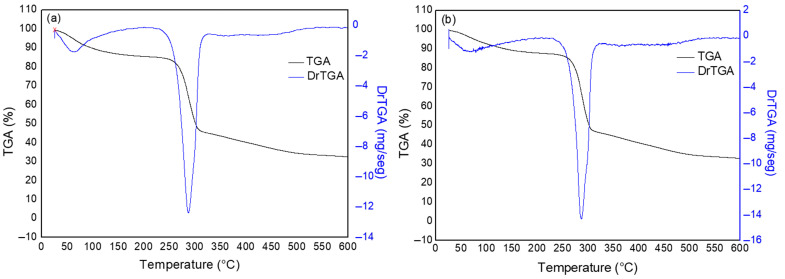
TGA and DrTGA curves for CMC LV (**a**) and CMC HV (**b**) powder samples.

Figure 3 shows the curves obtained from TGA and DrTGA for powder samples of CMC LV and CMC HV.

The first stage identified for the curve of CMC LV is in the range from 25 °C to 229 °C, with a mass loss of 13%. For CMC HV, this stage is identified between 26 °C and 230 °C, with a mass loss of 12%. This mass loss is attributed to dehydration, due to the loss of water molecules adsorbed in the hydrophilic chains of the polymer [34,35].

The second stage, identified in the curves for both samples, occurred in the range from 229 °C to 325 °C with a mass loss of 40% for CMC LV, and from 230 °C to 322 °C with a mass loss of 41% for CMC HV. This stage may be associated with the breaking of weak bonds of functional groups and the subsequent breaking of the cellulose chain into smaller units [36]. As a result, the sharp drop in mass is associated with polymer degradation, resulting from depolymerization and thermal decomposition reactions. These processes result in the formation of volatile products of low molecular weight. Due to the presence of COO2- groups in the CMC structure, decarboxylation occurs in this temperature range [37].

The last stage presented in the analysis occurred in the range from 325 °C to 600 °C with 12% mass loss for CMC LV, and from 335 to 600 °C with 13% mass loss for CMC HV. According to Britto and Assis (2009) [38], the gases generated by cellulose pyrolysis consist mainly of H_2_, CO_2_, CO, CH_4_, C_2_H_6_, C_2_H_4_, traces of larger gaseous organic compounds, and water vapor. This shows that at this stage, many chemical reactions occur, and the composition of the sample changes, as the CMC fragments reorganize during the thermal degradation process and the content of carbonized products increases [35]. The mass loss for this stage is therefore associated with the thermal degradation of dehydrated products of the carbon structure, including a wide range of alkanes, alkenes, dienes, and aromatic cyclization of carbonaceous residues [36].

**Figure 4:**
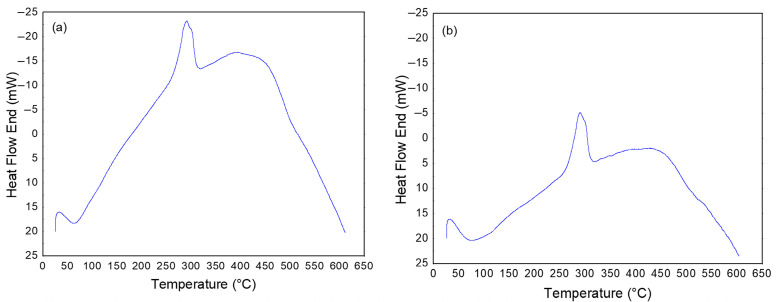
Thermal event curves for powder samples of CMC LV (**a**) and CMC HV (**b**).

Figure 4 shows the thermal events obtained by DSC for the LV and HV CMCs.

The results of the thermal events for CMC LV indicate the presence of an endothermic peak in the range between 36 °C and 132 °C, with an enthalpy of 277 J/g, attributed to the energy required for the elimination of water adsorbed in the sample. The second event, characterized by an exothermic peak, occurs between 273 °C and 309 °C, with an enthalpy of −229 J/g, associated with the thermal degradation of CMC. For CMC HV, the first endothermic event was observed in a temperature range similar to that of CMC LV, between 40 °C and 146 °C, with an enthalpy of 289 J/g, also attributed to the elimination of water. The second event, represented by an exothermic peak, was observed between 273 °C and 308 °C, with an enthalpy of −189 J/g, also related to the thermal degradation of CMC.

Like the TGA and DrTGA results (Figure 3), the DSC curves (Figure 4) also showed similarities regarding the thermal behavior of the CMC LV and HV samples. However, for the second event, which corresponds to sample degradation, CMC LV presents a higher enthalpy value. This fact may be related to the molecular structure of the polymer. Once CMC presents a lower viscosity, its structure facilitates the release of energy when compared to CMC HV and, therefore, characterizes it with greater difficulty in releasing energy [39]. The degradation range of CMCs is in agreement with the range of highest percentage of mass loss presented by the TGA curves (Figure 3) [37,40].

### 3.2. Characterization of CMC Solutions at Room Temperature

**Figure 5:**
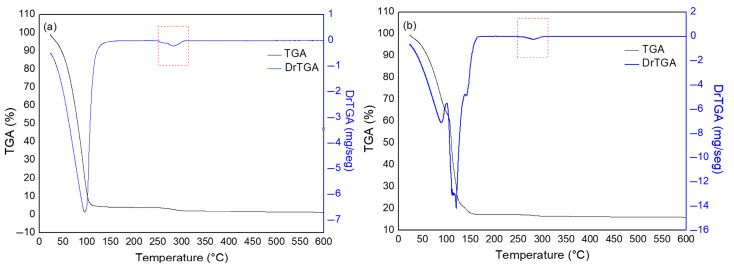
TGA and DrTGA curves for SFW (**a**) and SSW (**b**).

Figure 5 shows the curves obtained from TGA and DrTGA for the SFW and SSW solutions, in order to evaluate the influence of the presence of salt on the degradation of CMC.

According to the thermogravimetric curves, the thermal degradation of CMC in solution occurred in the range of 253 °C to 305 °C, with a mass loss of 1.7%, for SFW; and between 261 °C and 300 °C, with a loss of 0.6%, for SSW. These results indicate that the initial temperature of degradation of CMC increased by 22 °C for SFW and by 31 °C for SSW, compared to the onset temperature of CMC degradation in powder form.

The decomposition of CMC, observed in the identified thermal events, is closely related to the degradation of cellulose [41], the main component of the CMC molecular chain. However, it was expected that CMC in solution would have a degradation temperature range smaller than the degradation temperature interval for the powder state. This was attributed to the water’s capacity to autoionize under ambient conditions, forming pairs of hydronium and hydroxide ions in solution, whose activity would increase with the increase in temperature, shifting the chemical equilibrium of the water [42,43]. These ions can break the glycosidic bonds that join the repeating units (cellobiose) of the CMC chain, causing degradation of the material.

The smaller degradation of CMC in solution is related to the conditions of the thermogravimetric analysis test. Since the heating rate was 10 °C/min, even if the solution was exposed to high temperatures, the exposure time was short and, therefore, not sufficient for the degradation of CMC in solution to occur at lower temperatures. This was also observed in the study carried out by Plank and Gossen (1991) [44], who showed that complete degradation of CMC in solution only occurred after thermal aging at temperatures below 100 °C. Morais and collaborators (2018) described that, in general, the main chain of polymers is composed of carbon–carbon bonds, whose dissociation energy is high [45]. As such, this type of polymer is not easily depolymerizable, except when heated for long periods [45]. Thus, the results obtained with the CMC LV and CMC HV samples suggest that the degradation of CMC in solution is determined not only by temperature but also by the time of exposure of the CMC to temperature.

When comparing the two curves, it is also clear that the presence of salt resulted in changes in the curve pattern. This is denoted by a mass loss stage that is observed only in the TGA of SSW. For both solutions, the first mass loss stage is attributed to water evaporation. In the TGA of SFW, the interval occurred in a sharp drop in the curve, from 24 °C to 142 °C, with a mass loss of 94.42%. For SSW, the mass loss occurred in two intervals: one from 25 °C to 100 °C, with a mass loss of 35.7%, followed by a new drop in the curve in the interval from 100 °C to 139 °C, with a mass loss of 44%. The dissolved ions seem to interact with the water molecules through their hydroxyls through electrostatic forces. Ion-free water evaporates in the first temperature range, while water associated with ions is eliminated in the second temperature range. According to Rocha Filho and Silva (2023) [46], the presence of non-volatile solutes causes the boiling temperature of solutions to be higher than that of the pure liquid. Another possibility may be associated with the formation of complexes between NaCl and the CMC functional groups, which can delay water evaporation and/or make these complexes less volatile, causing them to be eliminated later. It is important to note that all of the water in the sample evaporates before reaching the CMC decomposition temperature, which indicates that the initial water and brine content does not affect the onset of the decomposition temperature [37].

**Figure 6:**
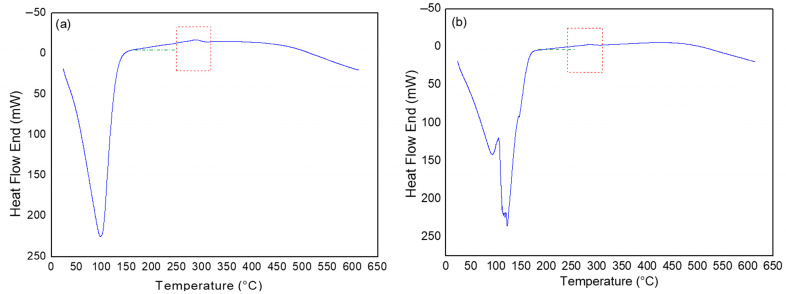
Thermal event curves obtained by DSC for SFW (**a**) and SSW (**b**) solutions.

Figure 6 shows the thermal events obtained by DSC for the SFW and SSW solutions.

Evaluating the curves presented in Figure 6, the thermal events are consistent with the results evaluated for the mass loss of the samples. For SFW, the first event occurs in the range from 38 °C to 126 °C, with an enthalpy value of 1875 J/g, characterized by an endothermic peak, which refers to the elimination of water. A second event appears in the range from 252 °C to 304 °C, with an enthalpy value of −11.7 J/g. For SSW, the first event is presented in the range from 54 °C to 103 °C, represented by an endothermic peak, reaching an enthalpy value of 132 J/g, related to the evaporation of water. The second event takes place between 110 °C and 140 °C; it shows an endothermic peak with an enthalpy value of 372 J/g. This event marks the end of the water elimination process, which may include molecules that were held by interactions with the salt.

In the range of approximately 150 °C to 250 °C, the curve is almost linear, suggesting that the sample neither absorbed nor released heat during this temperature range. This shows a higher thermal stability of CMC in solution when compared to CMC powder (Figure 4).

Comparing the results obtained for SFW and SSW, it can be seen that the presence of salt provides greater thermal stability in the range of 150 °C to 250 °C. For SFW, the water molecules form bridges between the CMC molecules, resulting in higher viscosity and, consequently, a greater need to absorb energy to initiate material degradation. In SSW, salt ions compete with water molecules for the carboxyl groups of CMC. This interaction increases the amount of free water, reduces viscosity in the system, and decreases the need for energy absorption.

**Figure 7:**
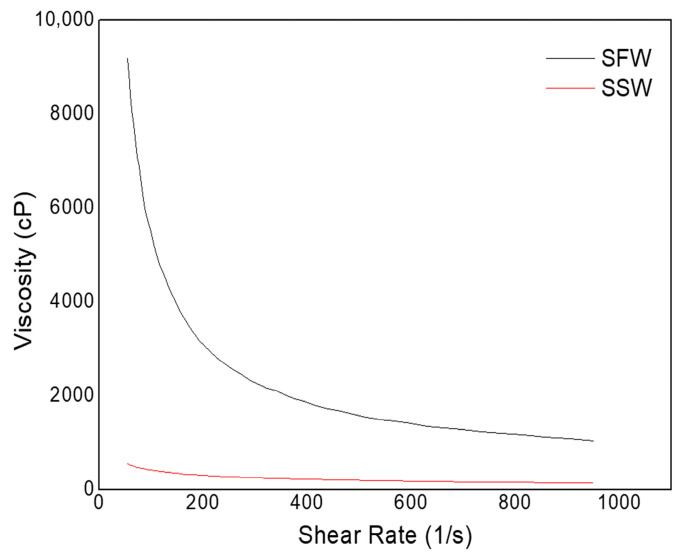
Viscosity of SFW and SSW at room temperature.

Figure 7 presents the viscosity results for SFW and SSW.

According to Figure 7, it is clear that SFW and SSW present distinct behaviors, showing that the presence of salt in the solution directly influences their rheological performance. The viscosity curves show a significant reduction in the viscosity values for SSW solution throughout the range of shear rates studied. The impact of salt interactions on the solutions’ rheology is also reflected in the flow parameters determined through the Ostwald–de Waele model fitting (Table 4).

The flow behavior of both solutions was accurately described by the Ostwald–de Waele model, as indicated by the R^2^ values ≥ 0.9. The SFW solution exhibited the lowest behavior index (n), at 0.19, while the SSW solution reached a value of 0.56, which indicates pseudoplastic behavior. The addition of salt to the solution resulted in a significant reduction in the consistency index (K), which decreased from 242.23 in the SFW solution to 3.10 in the SSW solution. These results confirm the impacts to the rheological behavior caused by the addition of NaCl to the CMC solution.

At the molecular level, Na^+^ and Cl^−^ ions interact with the system in two main ways: First, Na^+^ cations neutralize the anionic functional groups of CMC, reducing electrostatic repulsion and causing the polymer chains to coil, which decreases their hydrodynamic volume and, thus, lowers the solution’s viscosity. Second, Na^+^ binds to the oxygen atom of water molecules, and Cl^−^ to the hydrogen atoms, limiting water’s availability to interact with CMC. Without this water bridging, the formation of a tight polymer network is hindered, further reducing the viscosity.

Thus, although the presence of salt does not influence the thermal degradation of CMCs, the adjustment of rheological properties must consider the reduction in viscosity in the presence of electrolytes, making it necessary to adjust the polymer content according to the salinity required for the fluid.

### 3.3. Characterization of Saline Solution (SSW) After Exposure to Temperature

Given the similarity of the CMC degradation stage in SFW and SSW, the thermal degradation study, using a factorial design, was carried out only for SSW, since in most cases it is necessary to add brines to obtain the required density in the fluid formulations used in the oil industry.

**Figure 8:**
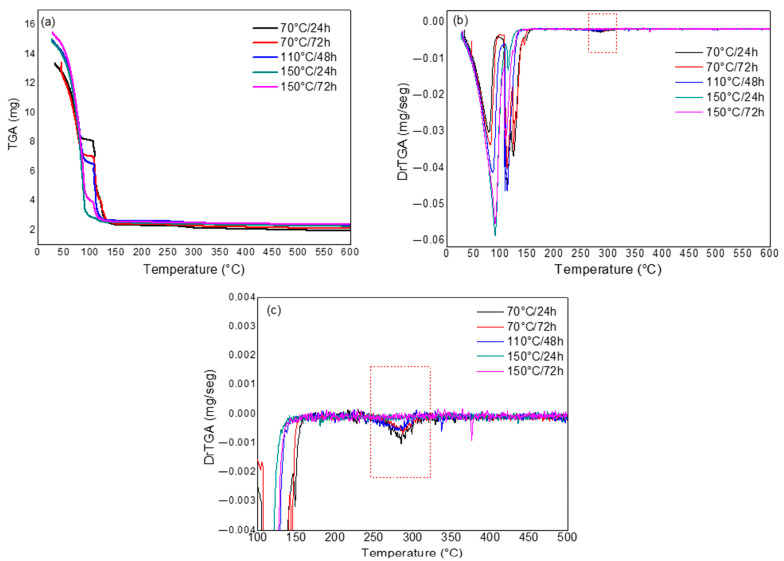
Thermogravimetric analysis of SSW exposed to different temperatures and times: TGA (**a**), DrTGA (**b**), and area corresponding to the presence or absence of the characteristic peak of CMC degradation on DrTGA (**c**).

Figure 8 presents the results obtained from the thermal analysis of this solution, exposed to different temperature and time conditions. The condition of 110 °C/48 h, which was the central point of the planning, was performed in triplicate. However, only one of the curves of this condition is represented in the graph.

According to Figure 8a, regardless of the exposure condition, up to 150 °C, the curves indicate two stages associated with the elimination of water from the solution, with values varying between 81% and 83%.

In Figure 8c, stages that occur in the temperature range equivalent to that observed for CMC degradation, i.e., in the range of approximately 220 °C to 320 °C, are highlighted. Thus, once this thermal stage is identified, it is possible to infer that the CMC in the solution was not completely degraded during the previous exposure to temperature, so the mass loss calculated in the thermal analysis for this event will correspond to the percentage of CMC that remains in solution. On the other hand, the absence of this stage implies that the CMC was degraded during the exposure of the solution to temperature.

When comparing the curves presented in Figure 8c, it can be observed that the CMC did not undergo degradation when exposed to 70 °C/24 h, 70 °C/72 h, and 110 °C/48 h, since the characteristic degradation stage is present in the curves. In the case of exposure of the solution to 150 °C/24 h and 150 °C/72 h, this stage is not identified, showing that the CMC was completely degraded when previously subjected to these conditions.

The mass loss values and temperature ranges corresponding to the characteristic stage of CMC degradation, for the tests performed according to the factorial design, are presented in Table 5.

According to Table 5, it can be seen that the greatest variations in the mass loss values are related to the change in temperature, regardless of the time used, so that for the highest temperature (150 °C), the CMC is completely degraded before the thermal analysis test, with no mass loss being recorded for the test in this case. Thus, this result suggests that, for the temperature and time conditions evaluated, the variable with the greatest influence on the degradation of the CMC is the temperature.

**Figure 9:**
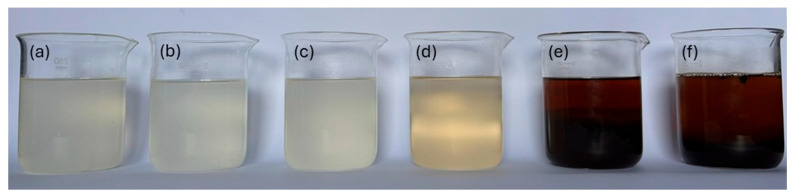
Images of CMC LV and HV solutions at room temperature (**a**), and exposed to 70 °C/24 h (**b**), 70 °C/72 h (**c**), 110 °C/48 h (**d**), 150 °C/24 h (**e**), and 150 °C/72 h (**f**).

Figure 9 shows images of the solution before and after exposure to different temperature and time conditions.

Based on the images presented, it can be seen that the solutions exposed to 70 °C/24 h and 70 °C/72 h present a whitish coloration, with a predominant physical aspect similar to that of the solution at room temperature. This result suggests the thermal stability of the CMC under these conditions, according to the data obtained in the thermal analysis. The first significant visual change was observed in the solution exposed to 110 °C/48 h, which presented a yellowish shade, possibly indicating an initial process of CMC degradation. For the images of the solution subjected to 150 °C/24 h and 150 °C/72 h, the dark coloration suggests complete degradation of the CMC, in accordance with the results of the thermal analysis of the sample.

In order to better assess the color differences, each solution was analyzed using the platinum–cobalt (Pt–Co) scale, which ranges from 0 (no color) to 500 (intense yellow color). The results obtained are presented in Table 6, according to the methodology described by Lipps et al. (2023) [47].

The colorimetric results presented in Table 6 support the visual evidence illustrated in Figure 9. The solutions exposed to 70 °C for 24 h and 70 °C for 72 h showed values of 126 and 128 mg Pt–Co/L, respectively, which are consistent with the mean color value of the SSW solution not subjected to thermal exposure (127 mg Pt–Co/L). When the exposure temperature was increased to 110 °C for 48 h, the value rose to 153 mg Pt–Co/L, confirming the occurrence of color changes between the solutions. Finally, the solutions exposed to 150 °C for 24 h and 150 °C for 72 h exhibited more pronounced changes, reaching values exceeding 500 mg Pt–Co/L.

From the results presented in Table 5, the mathematical model for the CMC mass loss was obtained from statistical regression, considering only the respective significant variables, at the 95% confidence level, presented in Equation (1). Table 7 presents the relevant statistics for the statistical analysis of variance (ANOVA) of this parameter.

ML = (0.77 ± 0.05) − (1.44T ± 0.14)(1)

where ML represents the mass loss and T the temperature.

The mathematical model demonstrates that only the temperature variable exerts a statistically significant influence on the CMC mass loss, and this relation is inversely proportional. It is worth reiterating that, in this situation, lower mass loss values indicate that a greater proportion of the polymer was previously degraded during exposure to temperature, and the expected mass loss value in the test corresponds to the fraction of mass remaining in solution at the time of thermal analysis. The coefficient of determination of 97.41% indicates that the quality of the adjustment of the equation to the variables analyzed is satisfactory, and the value obtained for the F test shows that this ratio is statistically significant and predictive at the 95% confidence level.

**Figure 10:**
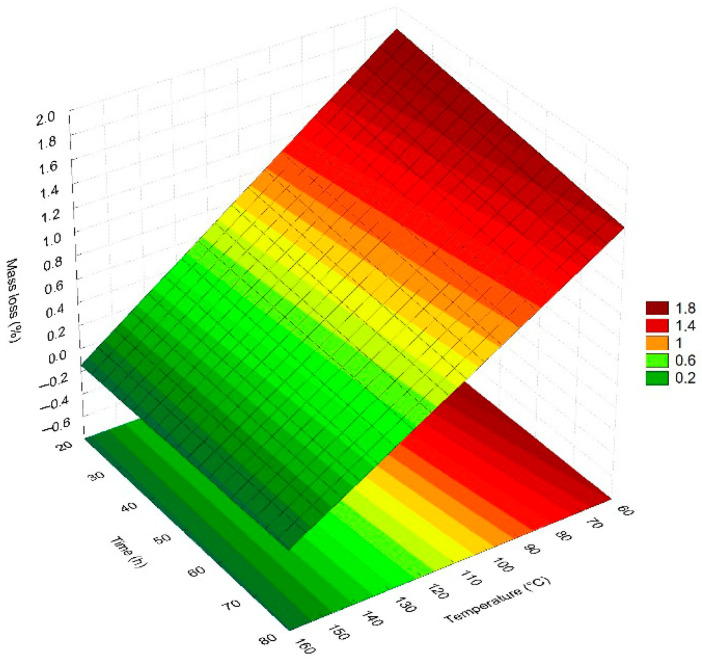
Response surface for the CMC mass loss in the SSW solution in relation to time and temperature.

The linear model presented in Equation (1) was used to obtain the response surface shown in Figure 10.

The response surface presented in Figure 10 confirms the correlation presented by the mathematical model; that is, the variable with the greatest influence on mass loss is temperature. Increasing temperature ensures a more significant degradation of CMC and results in a lower loss of residual mass to be degraded in thermogravimetric analysis tests.

**Figure 11:**
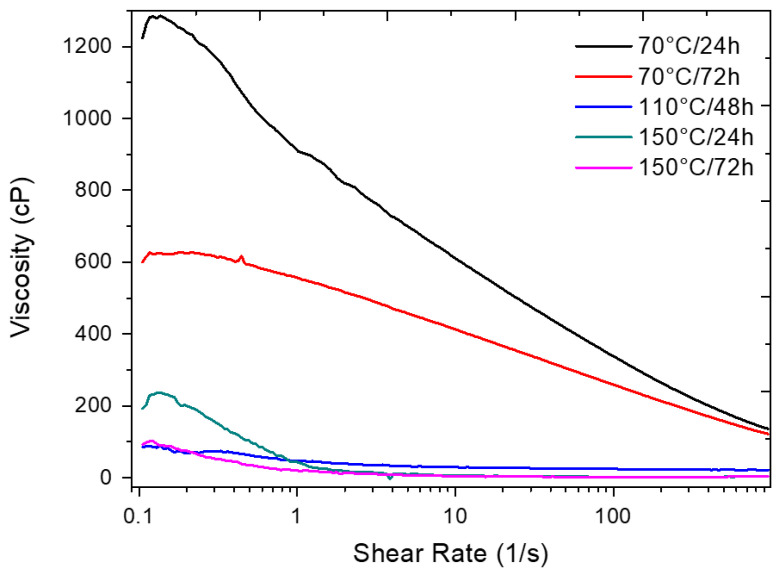
Viscosity curves as a function of shear rate of SSW after exposure to different temperatures and times.

Figure 11 presents the results of the viscosity curve as a function of the shear rate of the SSW solution exposed to different temperature and time conditions.

From the viscosity curves, it can be seen that there is an abrupt decrease in the viscosity of the exposed solution from 110 °C/48 h. Although the solution’s rheological properties were considerably impacted, according to the results of the thermal analyses, the CMC had not yet degraded under this condition. This indicates that the increase in temperature broke the polymer network, breaking the secondary bonds, resulting in repulsion between the molecules and, consequently, in a decrease in viscosity. For the conditions of 150 °C/24 h and 150 °C/72 h, the decrease of viscosity is directly related to the total degradation of the CMC. These results were also consistent with the flow parameters obtained by fitting the viscosity curves to the Ostwald–de Waele rheological model, presented in Table 8, which shows that the highest values for the consistency index (K) were obtained for the solutions exposed to 70 °C, with values of 1.90 and 1.09 for the 70 °C/24 h and 70 °C/72 h conditions, respectively. The solutions subjected to 150 °C/24 h and 150 °C/72 h exhibited K values of 0.00, indicating a complete loss of shear resistance, and suggesting total degradation of the CMC structure

For applications in the formulation of fluids for the oil industry, the results of this stage show that, in addition to the temperature and operating time, it is also necessary to consider the correlation between the thermal degradation of the polymer in solution and the viscosity results, since, for certain temperatures, even if there is no degradation of the CMC, it may be possible to observe the structural changes caused by exposure to temperature that result in compromising the activity of the polymer as a rheological agent and filtrate controller.

## 4. Conclusions

After evaluating the thermal degradation of carboxymethyl cellulose in solution in the presence of salt, as well as investigating the correlation of the rheological behavior of these solutions with exposure to temperature, we can conclude the following:

- There are no significant differences between the morphological and thermal characteristics of CMC LV and CMC HV in the powder state;
- The interaction of CMC with the salt present in solution has no significant influence on thermal degradation, but it directly influences the rheological behavior of the solution at room temperature;
- The SSW solution, composed of CMC LV and CMC HV solubilized in brine, remained thermally stable after exposure to conditions of 70 °C/24 h, 70 °C/12 h, and 110 °C/48 h, but it underwent total degradation at 150 °C/24 h and 150 °C/72 h;
- CMC LV and CMC HV, used together in saline solutions, may compromise the rheological behavior, even if there is no thermal degradation of the polymers, so that significant reductions in the viscosity values of the solutions are observed for temperatures above 110 °C.

In general, considering the application in fluids for the oil industry, it can be concluded that temperature exerts a significant influence on the degradation of CMC. The interaction of this variable with the exposure time does not demonstrate significant impacts, which ensures that the use of CMC in formulations of fluids applied in the oil industry should mainly consider the temperature conditions in wells, regardless of the operating time. However, it is suggested to study longer periods of time, in order to elucidate questions related to prolonged operations. In addition, it is evident that the application of fluids to high-temperature wells must be conditioned to the combined analysis of thermal degradation and rheological tests, since the thermal effects on the polymer structure are preliminary to the degradation stage, causing impacts on the viscosity of the fluid that can compromise its performance.

## Figures and Tables

**Table 1:** Identification and characteristics of the samples used in the work methodology.

Step	Samples	Physical State of the Sample	Tests Performed
1	CMC HV/CMC LV	Powder	SEM, FTIR, TGA/DrTGA, DSC
2	CMC HV + CMC LV	Solution (SFW and SSW)	TGA/DrTGA, DSC, and rheological tests
3	CMC HV + CMC LV	Solution (SSW)	Previous exposure to temperature (factorial design) + TGA/DrTGA and rheological tests

**Table 2:** Coded and actual levels of SSW solution temperature exposure parameters.

Variable	−1	0	+1
Temperature	70 °C	110 °C	150 °C
Exposure Time	24 h	48 h	72 h

**Table 3:** Planning matrix for exposure of solutions to temperature.

Test	Temperature	Time	Condition
1	−1	−1	70 °C/24 h
2	−1	+1	70 °C/72 h
3	+1	−1	150 °C/24 h
4	+1	+1	150 °C/72 h
5	0	0	110 °C/48 h
6	0	0	110 °C/48 h
7	0	0	110 °C/48 h

**Table 4:** Rheological indices obtained for SFW and SSW viscosity curves.

Solutions	R^2^	K (cP)	n
SFW	0.97	242.23	0.19
SSW	0.99	3.10	0.56

**Table 5:** Comparative values of mass loss and temperature ranges corresponding to the characteristic stage of CMC degradation.

Exposure Time and Temperature	Mass Loss (%)	Temperature Range (°C)	Polymer Degradation During Exposure to Temperature
70 °C/24 h	1.55	221–317	No
70 °C/72 h	1.33	225–321	No
110 °C/48 h	0.80	232–309	No
110 °C/48 h	0.98	240–303	No
110 °C/48 h	0.74	251–308	No
150 °C/24 h	*	27–125	Yes
150 °C/72 h	*	29–133	Yes

* No mass loss identified.

**Table 6:** Color of solutions according to Pt–Co scale.

Condition of Temperature/Time	Color (mq Pt–Co/L)
Room Temperature	127
70 °C/24 h	126
70 °C/72 h	128
110 °C/48 h	153
150 °C/24 h	>500
150 °C/72 h	>500

**Table 7:** Analysis of variance (ANOVA) for CMC mass loss in solution.

**Coefficient of Determination (R^2^)**	97.41%
**F_calculated_/F_tabulated_**	25.02

**Table 8:** Rheological indices obtained for SSW (after exposure to temperature) viscosity curves.

Temperature/ Exposure Time	R^2^	K (cP)	n
70 °C/24 h	0.99	1.90	0.62
70 °C/72 h	0.99	1.09	0.68
110 °C/48 h	0.99	0.03	0.96
150 °C/24 h	0.99	0.00	1.28
150 °C/72 h	0.99	0.00	1.51

## Data Availability

Data are contained within the article.
